# 3-[3-(3-Fluoro­phen­yl)-1,2,4-oxadiazol-5-yl]propionic acid

**DOI:** 10.1107/S1600536808042001

**Published:** 2008-12-17

**Authors:** Suseanne K. M. Santos, Ricardo A. W. Neves Filho, Adailton J. Bortoluzzi, Rajendra M. Srivastava

**Affiliations:** a Depto. de Química Fundamental, Universidade Federal de Pernambuco, 50740-540 Recife, Pernambuco, Brazil; b Depto. de Química, Universidade Federal de Santa Catarina, 88040-900 Florianópolis, Santa Catarina, Brazil

## Abstract

In the title compound, C_11_H_9_FN_2_O_3_, the benzene ring is almost coplanar with the heterocyclic ring, making a dihedral angle of 14.0 (1)°. The plane of the carboxyl group is rotated by 14.7 (3)° with respect to the 1,2,4-oxadiazole ring plane. The aliphatic chain exhibits a standard zigzag arrangement. Two inter­molecular O—H⋯O hydrogen bonds between the carboxyl groups related by an inversion centre promote a dimeric structure formation. The dimers are stacked along the crystallographic *a* axis.

## Related literature

For general background, see: Gallardo *et al.* (2008 ▶); Jakopin & Dolenc (2008 ▶). For related structures, see: Wang *et al.* (2006 ▶, 2007 ▶); Yan, Xing *et al.* (2006 ▶); Yan *et al.* (2006*a* ▶,*b* ▶). For the method of preparation, see: Sindkhedkar *et al.* (2008 ▶); Srivastava & Seabra (1997 ▶).
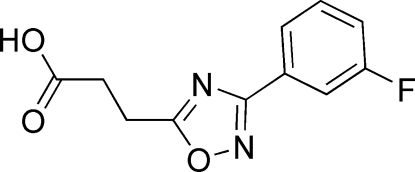


## Experimental

### 

#### Crystal data

C_11_H_9_FN_2_O_3_
*M* *_r_* = 236.20
Triclinic, 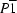

*a* = 5.055 (1) Å
*b* = 5.905 (1) Å
*c* = 17.967 (1) Å
α = 85.769 (5)°
β = 87.965 (7)°
γ = 81.252 (7)°
*V* = 528.47 (14) Å^3^
*Z* = 2
Mo *K*α radiation
μ = 0.12 mm^−1^
*T* = 293 (2) K
0.50 × 0.33 × 0.07 mm

#### Data collection

Enraf–Nonius CAD-4 diffractometer
Absorption correction: none
2136 measured reflections
2066 independent reflections
1557 reflections with *I* > 2σ(*I*)
*R*_int_ = 0.010
3 standard reflections every 200 reflections intensity decay: 1%

#### Refinement

*R*[*F* ^2^ > 2σ(*F* ^2^)] = 0.039
*wR*(*F* ^2^) = 0.112
*S* = 1.06
2066 reflections
158 parameters
H atoms treated by a mixture of independent and constrained refinement
Δρ_max_ = 0.21 e Å^−3^
Δρ_min_ = −0.21 e Å^−3^

### 

Data collection: *CAD-4 Software* (Enraf–Nonius, 1989 ▶); cell refinement: *CAD-4 Software*; data reduction: *HELENA* (Spek, 1996 ▶); program(s) used to solve structure: *SIR97* (Altomare *et al.*, 1999 ▶); program(s) used to refine structure: *SHELXL97* (Sheldrick, 2008 ▶); molecular graphics: *PLATON* (Spek, 2003 ▶) and *Mercury* (Macrae *et al.*, 2006 ▶); software used to prepare material for publication: *SHELXL97*.

## Supplementary Material

Crystal structure: contains datablocks global, I. DOI: 10.1107/S1600536808042001/is2367sup1.cif

Structure factors: contains datablocks I. DOI: 10.1107/S1600536808042001/is2367Isup2.hkl

Additional supplementary materials:  crystallographic information; 3D view; checkCIF report

## Figures and Tables

**Table 1:** Hydrogen-bond geometry (Å, °)

*D*—H⋯*A*	*D*—H	H⋯*A*	*D*⋯*A*	*D*—H⋯*A*
O10—H10⋯O9^i^	0.97 (3)	1.68 (3)	2.650 (2)	179 (3)

Symmetry code: (i) 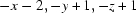.

**Fig. 1.**
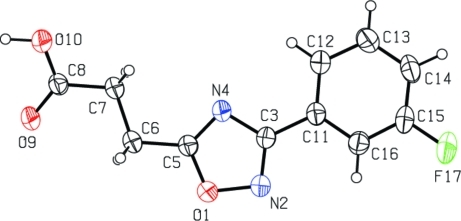
The molecular structure of (I) with labeling scheme. Displacement ellipsoids are shown at the 40% probability level.

**Fig. 2.**
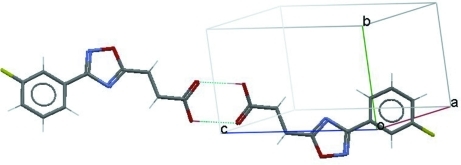
Dimeric strucuture formed by hydrogen bonding.

**Fig. 3.**
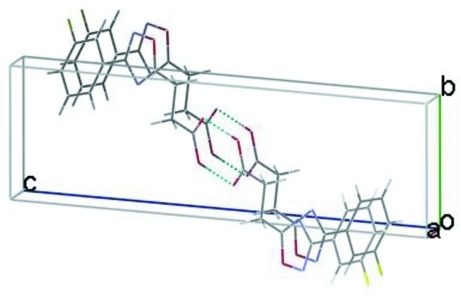
Molecules of (I) stacked along the a axis.

## supplementary crystallographic information

### Comment

1,2,4-Oxadiazoles are well known compounds, which exhibit a large number of biological activities (Jakopin & Dolenc, 2008). Recently, the use of this heterocycle as core for luminescent liquid crystals has also been described (Gallardo *et al.*, 2008).

In the title compound (Fig. 1), the bond lengths and angles are in agreement with the values previously reported for 1,2,4-oxadiazole-containing molecules (Wang *et al.*, 2006, 2007; Yan, Xing *et al.*, 2006; Yan *et al.*,  2006*a*,*b*). The torsion angle N2—C3—C11—C16 between the benzene ring attached to C-3 of the 1,2,4-oxadiazole system is -13.6 (2)°, thus, both rings are almost coplanar. The C-5 side-chain containing a carboxylic acid group shows a zigzag arrangement, having the torsion angle C5—C6—C7—C8 of -179.4 (1)°. In addition, the plane of the carboxylic group is also rotated by 14.7 (3)° with respect to the mean plane of the 1,2,4-oxadiazole five-membered ring, but in opposite direction of deviation of the fluoro-phenyl ring. This makes the molecular structure to be slightly twisted. Carboxylic groups are involved in centrosymmetric intermolecular hydrogen-bonding forming a dimeric structure (Fig. 2). The dimmers are perfectly stacked along the crystallographic *a* axis (Fig. 3).

### Experimental

The title compound was synthesized following the procedure reported earlier for the analogous compounds (Srivastava & Seabra, 1997; Sindkhedkar *et al.*, 2008). A mixture of 3-fluorbenzamidoxime (2.0 mmol) and succinic anhydride (2.2 mmol) was heated in a domestic microwave oven for 10 min. The crude material was purified by column chromatography. Crystallization of pure material from chloroform, from which a suitable crystal was chosen for the X-ray crystallographic experiment.

### Refinement

H atoms attached to C atoms were added at their calculated positions and included in the structure factors calculations, with C—H = 0.93 (aromatic) and 0.97 Å (methylene), and with *U*_iso_(H) = 1.2*U*_eq_(C). The H atom of carboxylic acid was located in a difference Fourier map and treated as a free atom.

### Crystal data

**Table:**

C_11_H_9_FN_2_O_3_	*Z* = 2
*M**_r_* = 236.20	*F*(000) = 244
Triclinic, *P*1	*D*_x_ = 1.484 Mg m^−^^3^
Hall symbol: -P 1	Mo *K*α radiation, λ = 0.71069 Å
*a* = 5.055 (1) Å	Cell parameters from 25 reflections
*b* = 5.905 (1) Å	θ = 5.0–18.8°
*c* = 17.967 (1) Å	µ = 0.12 mm^−^^1^
α = 85.769 (5)°	*T* = 293 K
β = 87.965 (7)°	Irregular plate, colorless
γ = 81.252 (7)°	0.50 × 0.33 × 0.07 mm
*V* = 528.47 (14) Å^3^	

### Data collection

**Table:**

Enraf–Nonius CAD-4 diffractometer	*R*_int_ = 0.010
Radiation source: fine-focus sealed tube	θ_max_ = 26.0°, θ_min_ = 1.1°
graphite	*h* = −6→6
ω–2θ scans	*k* = −7→7
2136 measured reflections	*l* = −22→0
2066 independent reflections	3 standard reflections every 200 reflections
1557 reflections with *I* > 2σ(*I*)	intensity decay: 1%

### Refinement

**Table:**

Refinement on *F*^2^	Primary atom site location: structure-invariant direct methods
Least-squares matrix: full	Secondary atom site location: difference Fourier map
*R*[*F*^2^ > 2σ(*F*^2^)] = 0.039	Hydrogen site location: inferred from neighbouring sites
*wR*(*F*^2^) = 0.112	H atoms treated by a mixture of independent and constrained refinement
*S* = 1.06	*w* = 1/[σ^2^(*F*_o_^2^) + (0.0583*P*)^2^ + 0.0868*P*] where *P* = (*F*_o_^2^ + 2*F*_c_^2^)/3
2066 reflections	(Δ/σ)_max_ < 0.001
158 parameters	Δρ_max_ = 0.21 e Å^−^^3^
0 restraints	Δρ_min_ = −0.21 e Å^−^^3^

### Fractional atomic coordinates and isotropic or equivalent isotropic displacement parameters (Å^2^)

**Table:**

	*x*	*y*	*z*	*U*_iso_*/*U*_eq_	
C3	0.1073 (3)	−0.0359 (3)	0.24108 (9)	0.0403 (4)	
C5	−0.1973 (3)	0.0022 (3)	0.32245 (9)	0.0412 (4)	
C6	−0.4105 (3)	0.0731 (3)	0.37879 (10)	0.0463 (4)	
H6A	−0.3429	0.0278	0.4284	0.056*	
H6B	−0.5616	−0.0064	0.3722	0.056*	
C7	−0.5043 (3)	0.3291 (3)	0.37270 (10)	0.0476 (4)	
H7A	−0.3529	0.4086	0.3788	0.057*	
H7B	−0.5738	0.3741	0.3233	0.057*	
C8	−0.7169 (3)	0.4009 (3)	0.42998 (9)	0.0422 (4)	
C11	0.3022 (3)	0.0236 (3)	0.18257 (9)	0.0415 (4)	
C12	0.2778 (4)	0.2461 (3)	0.15009 (10)	0.0522 (4)	
H12	0.1426	0.3584	0.1660	0.063*	
C13	0.4575 (4)	0.2996 (4)	0.09349 (11)	0.0618 (5)	
H13	0.4413	0.4485	0.0715	0.074*	
C14	0.6588 (4)	0.1352 (4)	0.06958 (11)	0.0594 (5)	
H14	0.7785	0.1708	0.0316	0.071*	
C15	0.6783 (3)	−0.0823 (3)	0.10322 (10)	0.0534 (5)	
C16	0.5063 (3)	−0.1432 (3)	0.15924 (10)	0.0483 (4)	
H16	0.5256	−0.2923	0.1811	0.058*	
N2	0.0849 (3)	−0.2473 (3)	0.26222 (9)	0.0552 (4)	
N4	−0.0667 (3)	0.1270 (2)	0.27700 (8)	0.0437 (3)	
O1	−0.1230 (2)	−0.2237 (2)	0.31750 (7)	0.0555 (4)	
O9	−0.8599 (2)	0.2685 (2)	0.45969 (7)	0.0547 (3)	
O10	−0.7395 (3)	0.6168 (2)	0.44456 (8)	0.0551 (3)	
F17	0.8776 (2)	−0.2448 (2)	0.07982 (7)	0.0822 (4)	
H10	−0.885 (6)	0.660 (5)	0.4800 (16)	0.102 (8)*	

### Atomic displacement parameters (Å^2^)

**Table:**

	*U*^11^	*U*^22^	*U*^33^	*U*^12^	*U*^13^	*U*^23^
C3	0.0353 (8)	0.0424 (8)	0.0424 (8)	−0.0037 (6)	0.0030 (7)	−0.0040 (7)
C5	0.0372 (8)	0.0418 (8)	0.0440 (9)	−0.0059 (6)	0.0037 (7)	−0.0008 (7)
C6	0.0424 (9)	0.0479 (9)	0.0476 (9)	−0.0091 (7)	0.0116 (7)	0.0011 (7)
C7	0.0432 (9)	0.0465 (9)	0.0510 (10)	−0.0062 (7)	0.0137 (7)	0.0019 (7)
C8	0.0359 (8)	0.0454 (9)	0.0442 (9)	−0.0060 (7)	0.0050 (7)	0.0014 (7)
C11	0.0373 (8)	0.0482 (9)	0.0402 (8)	−0.0093 (7)	0.0040 (6)	−0.0076 (7)
C12	0.0533 (10)	0.0504 (10)	0.0511 (10)	−0.0047 (8)	0.0130 (8)	−0.0059 (8)
C13	0.0734 (13)	0.0582 (12)	0.0543 (11)	−0.0167 (10)	0.0160 (9)	−0.0023 (9)
C14	0.0573 (11)	0.0746 (14)	0.0489 (10)	−0.0210 (10)	0.0186 (9)	−0.0092 (9)
C15	0.0414 (9)	0.0669 (12)	0.0518 (10)	−0.0044 (8)	0.0114 (8)	−0.0165 (9)
C16	0.0445 (9)	0.0497 (10)	0.0503 (10)	−0.0057 (7)	0.0045 (8)	−0.0070 (7)
N2	0.0529 (9)	0.0452 (8)	0.0633 (10)	−0.0008 (6)	0.0206 (7)	0.0004 (7)
N4	0.0412 (7)	0.0429 (7)	0.0468 (8)	−0.0076 (6)	0.0108 (6)	−0.0045 (6)
O1	0.0560 (7)	0.0418 (7)	0.0646 (8)	−0.0033 (5)	0.0208 (6)	0.0038 (5)
O9	0.0488 (7)	0.0519 (7)	0.0636 (8)	−0.0129 (5)	0.0229 (6)	−0.0052 (6)
O10	0.0517 (7)	0.0471 (7)	0.0665 (8)	−0.0103 (5)	0.0198 (6)	−0.0077 (6)
F17	0.0635 (7)	0.0919 (10)	0.0841 (9)	0.0081 (6)	0.0308 (6)	−0.0145 (7)

### Geometric parameters (Å, °)

**Table:**

C3—N2	1.298 (2)	C11—C12	1.387 (2)
C3—N4	1.382 (2)	C11—C16	1.389 (2)
C3—C11	1.475 (2)	C12—C13	1.390 (3)
C5—N4	1.291 (2)	C12—H12	0.9300
C5—O1	1.338 (2)	C13—C14	1.376 (3)
C5—C6	1.486 (2)	C13—H13	0.9300
C6—C7	1.511 (2)	C14—C15	1.370 (3)
C6—H6A	0.9700	C14—H14	0.9300
C6—H6B	0.9700	C15—F17	1.359 (2)
C7—C8	1.497 (2)	C15—C16	1.370 (2)
C7—H7A	0.9700	C16—H16	0.9300
C7—H7B	0.9700	N2—O1	1.4183 (19)
C8—O9	1.2252 (19)	O10—H10	0.97 (3)
C8—O10	1.308 (2)		
			
N2—C3—N4	114.80 (14)	C12—C11—C3	119.61 (15)
N2—C3—C11	122.14 (14)	C16—C11—C3	120.18 (15)
N4—C3—C11	123.06 (14)	C11—C12—C13	119.36 (17)
N4—C5—O1	113.59 (14)	C11—C12—H12	120.3
N4—C5—C6	129.61 (15)	C13—C12—H12	120.3
O1—C5—C6	116.79 (13)	C14—C13—C12	120.87 (18)
C5—C6—C7	112.30 (13)	C14—C13—H13	119.6
C5—C6—H6A	109.1	C12—C13—H13	119.6
C7—C6—H6A	109.1	C15—C14—C13	118.25 (17)
C5—C6—H6B	109.1	C15—C14—H14	120.9
C7—C6—H6B	109.1	C13—C14—H14	120.9
H6A—C6—H6B	107.9	F17—C15—C14	118.33 (16)
C8—C7—C6	112.31 (14)	F17—C15—C16	118.68 (18)
C8—C7—H7A	109.1	C14—C15—C16	122.99 (17)
C6—C7—H7A	109.1	C15—C16—C11	118.33 (17)
C8—C7—H7B	109.1	C15—C16—H16	120.8
C6—C7—H7B	109.1	C11—C16—H16	120.8
H7A—C7—H7B	107.9	C3—N2—O1	102.97 (13)
O9—C8—O10	123.07 (15)	C5—N4—C3	102.40 (13)
O9—C8—C7	122.54 (15)	C5—O1—N2	106.23 (12)
O10—C8—C7	114.38 (14)	C8—O10—H10	112.6 (16)
C12—C11—C16	120.20 (15)		
			
N4—C5—C6—C7	8.6 (3)	C13—C14—C15—C16	0.2 (3)
O1—C5—C6—C7	−172.42 (15)	F17—C15—C16—C11	−179.68 (16)
C5—C6—C7—C8	−179.36 (14)	C14—C15—C16—C11	0.3 (3)
C6—C7—C8—O9	−23.4 (2)	C12—C11—C16—C15	−0.8 (3)
C6—C7—C8—O10	157.66 (15)	C3—C11—C16—C15	178.08 (15)
N2—C3—C11—C12	165.28 (17)	N4—C3—N2—O1	−0.10 (19)
N4—C3—C11—C12	−14.0 (2)	C11—C3—N2—O1	−179.48 (14)
N2—C3—C11—C16	−13.6 (2)	O1—C5—N4—C3	−0.79 (18)
N4—C3—C11—C16	167.08 (15)	C6—C5—N4—C3	178.25 (16)
C16—C11—C12—C13	0.7 (3)	N2—C3—N4—C5	0.54 (19)
C3—C11—C12—C13	−178.15 (17)	C11—C3—N4—C5	179.91 (15)
C11—C12—C13—C14	−0.2 (3)	N4—C5—O1—N2	0.77 (19)
C12—C13—C14—C15	−0.3 (3)	C6—C5—O1—N2	−178.40 (14)
C13—C14—C15—F17	−179.80 (17)	C3—N2—O1—C5	−0.37 (17)

### Hydrogen-bond geometry (Å, °)

**Table:**

*D*—H···*A*	*D*—H	H···*A*	*D*···*A*	*D*—H···*A*
O10—H10···O9^i^	0.97 (3)	1.68 (3)	2.650 (2)	179 (3)

Symmetry codes: (i) −*x*−2, −*y*+1, −*z*+1.
